# Clinical Performance of Three Commercial SARS‐CoV‐2 Rapid Antigen Tests for Community-Dwelling Individuals in a Tropical Setting

**DOI:** 10.3389/fcimb.2022.832235

**Published:** 2022-07-05

**Authors:** Diana Morales-Jadán, Carolina Viteri-Dávila, Bernardo Castro-Rodriguez, Alexander Paolo Vallejo-Janeta, Ismar A. Rivera-Olivero, Franklin Perez, Miguel Angel Garcia-Bereguiain

**Affiliations:** ^1^One Health Research Group, Universidad de las Américas, Quito, Ecuador; ^2^OneLabt, Santa Elena, Ecuador

**Keywords:** antigen test, RapiGEN^®^ Ag test kit, SD-Biosensor, Certest, SARS–CoV–2, clinical performance

## Abstract

During the second year of the COVID-19 pandemic, the use of Rapid Diagnosis Antigen Tests (RDAgTs) for SARS-CoV-2 detection has substantially increased as some of the brands available in the market were certified for clinical use by international regulatory agencies. RDAgTs are a fast and cheap tool for SARS-CoV-2 surveillance with great potential to improve testing capacities in middle- and low-income countries compared to the gold standard RT-qPCR. However, as the clinical performance of RDAgTs has been shown to vary greatly between the commercial brands available, evaluation studies are necessary. Moreover, the available evaluation has been done in high-income countries while SARS-CoV-2 transmission is also actively happening in developing countries, many of which are located in tropical latitudes where cross-reactivity with other infectious agents is highly prevalent, which could compromise RDAgT specificity. Moreover, unreported mutations and/or new SARS-CoV-2 variants may compromise RDAgT sensitivity as genomic surveillance is limited in these settings. Here we describe a multicenter and manufacturer‐independent evaluation of the clinical performance and analytical sensitivity of three different RDAgTs brands available in South America from three companies, Rapigen (South Korea), SD-Biosensor (South Korea), and Certest (Spain), compared to the gold standard RT-qPCR. A total number of 1,646 nasopharyngeal swabs from community-dwelling individuals were included in the study, and 379 of them were SARS-CoV-2 positive by RT-qPCR. The overall sensitivity for each RDAgT was 79% (IC95%: 72 - 86.2), 64.2% (IC95%: 56.7 - 71.6), and 45.8% (IC95%: 35.8 - 55.8) for SD-Biosensor, Certest, and Rapigen, respectively. The overall specificity for each RDAgT was 100%, 97.7% (IC95%: 96.8 - 98.6), and 100% for SD-Biosensor, Certest, and Rapigen, respectively. However, the limit of detection (LoD) to achieve a sensitivity over 90% was substantially lower for Certest RDAgT (10^2^ copies/uL) compared to SD-Biosensor (10^3^ copies/uL) or Rapigen (10^6^ copies/uL) RDAgTs, considering that the gold standard RT-qPCR method used in this study has a high sensitivity of 97.7% and low LoD of 5 copies/uL. Additionally, the Certest RDAgT also showed an improved sensitivity up to 79.7% (IC95%: 70.2 – 89.2) for symptomatic individuals. Finally, the slight reduction in specificity for Certest RDAgTs was only associated with one of the laboratories performing this study, pointing out the need for locally assessed evaluation for RDAgTs like this one carried out in Ecuador. In conclusion, two of the three the RDAgTs tested in this study are a fast, cheap, and point of care tool for SARS-CoV-2 surveillance and reliable enough to detect SARS-CoV-2 infectious individuals.

## Introduction

After the initial COVID-19 outbreak in Wuhan, China, in December 2019, SARS-CoV-2 spread rapidly and the World Health Organization declared COVID-19 a pandemic on 11 March 2020, and this pandemic is still ongoing (Wang et al., 2020; Gorbalenya et al., 2020; Zhou et al., 2020). SARS-CoV-2 RNA detection by RT-qPCR was the gold standard for acute infection diagnosis during the first year of the COVID-19 pandemic (Corman et al., 2020). By the end of 2020 and during 2021, the use of several commercial brands of point of care or Rapid Diagnosis Antigen Tests for SARS-CoV-2 detection became endorsed by international regulatory agencies or public health authorities (Cerutti et al., 2020; Pray et al., 2021). However, RT-qPCR is still widely used to confirm SARS-CoV-2 infection though this technique has several limitations for a scenario like the current COVID-19 pandemic: it is not easy to improve as a point of care diagnosis method, it requires sophisticated laboratory infrastructure, it depends on skilled personnel with a molecular biology background, and it is also permanently dependent on reagents that have experienced supply shortages (Freire-Paspuel et al., 2020; Cubas-Atienzar et al., 2021). Moreover, both RT-qPCR effectiveness for triage and contact tracing surveillance strategies are challenged by the need for 24 to 72 hours from sample collection to diagnosis (Kretzschmar et al., 2020). Additionally, RT-qPCR is an expensive diagnostic tool in the context of middle- and low-income countries that compromise their testing capacities (Cuellar et al., 2021; Santander-Gordon et al., 2021).

By contrast, the lateral flow immunoassays for SARS-CoV-2 antigen detection, also known as Rapid Diagnosis Antigen Tests (RDAgTs), allow for the point of care identification of SARS-CoV-2 virus in nasopharyngeal, oropharyngeal, or nasal samples in a time frame of 10 to 30 minutes depending on the commercial brand (Cerutti et al., 2020; Cubas-Atienzar et al., 2021; Iglὁi et al., 2021; Lee et al., 2021; Pray et al., 2021). Moreover, RDAgTs can either be performed by nursing staff without any laboratory infrastructure requirements or have been validated for patient self diagnosis (Nagura-Ikeda et al., 2020; Marx et al., 2021). Additionally, the cost of RDAgTs diagnosis is substantially cheaper than RT-qPCR diagnosis, as there are currently several RDAgTs commercial brands for self diagnosis sold for less than 5 USD even at grocery stores in the USA and some European countries. As RDAgTs are cheaper, faster, and available for point of care diagnosis, they are a powerful tool for SARS-CoV-2 surveillance, not only for triage in hospital settings for symptomatic individuals but also for the massive screening of community-dwelling individuals in middle- and low-income countries (Iglὁi et al., 2021; Marx et al., 2021; Pollock et al., 2021; Pray et al., 2021; Tinker et al., 2021).

Studies have addressed the clinical performance of different RDAgT brands compared to the gold standard RT-qPCR (Cubas-Atienzar et al., 2021; Lee et al., 2021). Those studies confirm that RDAgTs have reduced sensitivity and a higher limit of detection compared to RT-qPCR (Cubas-Atienzar et al., 2021; Lee et al., 2021). However, the accuracy of some RDAgTs brands has been suggested to allow the identification of the vast majority of infectious individuals, as the sensitivity is over 90% for viral loads with > 10^6^ genomic virus copies/ml (Corman et al., 2021; Cubas-Atienzar et al., 2021; Lee et al., 2021). Additionally, under a scenario like the COVID-19 pandemic, a reduction in sensitivity is acceptable as long as it comes with an increase in testing capacities, so the final output is a higher number of SARS-CoV-2 positive individuals detected (Mina et al., 2020). RDAgTs would fulfill these requirements as they are fast, cheap, and accurate enough to allow massive and rapid detection and isolation of new cases to stop transmission chains and reduce the impact of COVID-19 (World Health Organization (WHO), 2020; Andreani et al., 2021; Corman et al., 2021; Cubas-Atienzar et al., 2021; Lee et al., 2021; Pekosz et al., 2021; Weiss and Bellmann-Weiler, 2021).

As we have described above, the SARS-CoV-2 testing capacity in developing countries has been a challenge during the COVID-19 pandemic as it has been relying on the RT-qPCR technique. Moreover, as vaccination programs have been progressing slowly in middle- and low-income countries, SARS-CoV-2 circulation is still happening very actively in those settings, threatening COVID-19 pandemic control and eradication through new SARS-CoV-2 variant appearances (Dhawan et al., 2022). RDAgTs have the necessary features to improve effective SARS-CoV-2 surveillance programs in developing countries (World Health Organization (WHO), 2020; Andreani et al., 2021; Corman et al., 2021; Lee et al., 2021; Pekosz et al., 2021; Weiss and Bellmann-Weiler, 2021). However, the clinical performance evaluation studies for RDAgTs have been done in high-income countries (Albert et al., 2021; Andreani et al., 2021; Baro et al., 2021; Cerutti et al., 2020; Corman et al., 2021; Cubas-Atienzar et al., 2021; Iglὁi et al., 2021; Lee et al., 2021; Pérez-García et al., 2021; Pollock et al., 2021; Pray et al., 2021; Tinker et al., 2021; Weitzel et al., 2021). It has been already reported that low-quality COVID-19 diagnosis products are commercialized in developing countries and genomic surveillance in those settings is limited, so the tracking of new mutations or variants of SARS-CoV-2 potentially compromises the sensitivity of RDAgTs for COVID-19 diagnosis (Cota et al., 2020; Freire-Paspuel and Garcia-Bereguiain, 2020; Freire-Paspuel and Garcia-Bereguiain, 2021; Freire-Paspuel and Garcia-Bereguiain, 2021; Freire-Paspuel et al., 2021). Moreover, as the cross reactivity with other infectious pathogens for SARS-CoV-2 serology testing has been described, this phenomenon may also happen for RDAgTs, compromising their specificity in these middle- and low-income tropical countries (Echeverría et al., 2021; Faccini-Martínez et al., 2020; Tso et al., 2021). Considering this scenario, clinical performance evaluation of RDAgTs in the context of middle- and low-income countries are mandatory.

The aim of this work was to address the clinical performance and analytical sensitivity of three RDAgT commercial brands available to community-dwelling individuals in Ecuador.

## Materials and Methods

### Study Design

A total number of 1,646 community-dwelling individuals (COVID-19 asymptomatic or mildly symptomatic) were included in the study performed from 12 January to 8 May 2021 at two independent laboratories: 1,076 samples were taken at a laboratory for SARS-CoV-2 detection at “Universidad de Las Américas” in Quito, Pichincha province, Ecuador (UDLA lab); and 570 samples were taken at “OneLabt” laboratory in Ballenita, Santa Elena province, Ecuador. Overall, the study population included 1,267 individuals who tested negative and 379 who tested positive for SARS-CoV-2 detection by RT-qPCR (29.9% positivity rate).

A single nasopharyngeal swab was collected for each individual and tested for SARS-CoV-2 detection by RT-qPCR following the standard protocol in both laboratories. As the sample collection buffer volume was sufficient to perform RT-qPCR and RDAgTs, the spare sample volume was immediately processed for SARS-CoV-2 detection by RDAgT.

According to Ecuadorian regulations, all the results for SARS-CoV-2 detection made by RT-qPCR must be reported to the Ministry of Health, where a short survey is completed and information regarding COVID-19 related symptoms for individuals is stored. Based on this survey, we could classify our study groups as symptomatic or asymptomatic individuals.

### SARS-CoV-2 Detection Using Rapid Diagnosis Antigen Tests

Three different commercial brands of RDAgTs were evaluated in this study: Biocredit Covid-19 Ag Detection Kit (RapiGen, South Korea), SARS-CoV-2 Ag Test (Certest Biotec, Spain), and SARS-CoV-2 Rapid Antigen Test (SD-Biosensor, South Korea). Hereafter, we refer to the different test kits using the names “Rapigen”, “Certest”, and “SD-Biosensor”.

The three RDAgTs included in the study are based on lateral flow immunochromatography. We used the collection buffer provided for each RDAgT for sample collection and follow each manufacturer’s instructions to perform the SARS-CoV-2 detection. The reading time for the RDAgT varied from 10 to 30 min depending on the commercial brand.

As only one sample was collected from each patient, there were only paired samples for each RDAgT brand and RT-qPCR: 200 samples for Rapigen; 223 samples for SD-Biosensor; 1,223 for Certest. The variability or bias of the sample size for each commercial brand was due to the total number of RDAgTs that were kindly donated by each Ecuadorian distribution company for each of those brands. For Rapigen and “SD-Biosenseor, all the samples were processed at the UDLA lab. However, for the Certest evaluation, 653 and 570 samples were processed at UDLA lab and Onelabt, respectively.

### SARS-CoV-2 Detection Using RT-qPCR

Both laboratories involved in the study performed SARS-CoV-2 detection by RT-qPCR with the same protocol based on an adapted version from the Centers for Disease Control and Prevention (USA) protocol by using a CFX96 BioRad instrument and a triplex PCR assays (Freire-Paspuel et al., 2020; Freire-Paspuel and Garcia-Bereguiain, 2021; Freire-Paspuel et al., 2021; Freire-Paspuel et al., 2021). Briefly, the commercial kit ECUGEN SARS-CoV-2 RT-qPCR kit (UDLA-Startnewcorp, Ecuador) includes a triplex assay for N1 and N2 viral targets to detect SARS-CoV-2 and RNase P as an RNA extraction quality control (Freire-Paspuel and Garcia-Bereguiain, 2021). Also, negative controls (sample collection buffer) were included as a control for carry-over contamination, one for each set of RNA extractions. For viral loads calculation, the 2019-nCoV N positive control (IDT, USA) was used and provided at 200.000 genome equivalents/mL (Freire-Paspuel et al., 2020; Freire-Paspuel et al., 2021).

This positive control is a plasmid including N1 and N2 viral gene targets sequences, and it is a SARS-CoV-2 positive control recommended by CDC guidelines (Freire-Paspuel et al., 2020; Freire-Paspuel et al., 2021). Serial dilutions of the positive control were included in each set of samples RT-qPCR running, so an internal calibration curve with known concentrations of genomic SARS-CoV-2 material was always available. A regression analysis was made for each of those calibration curves taking RT-qPCR Ct values for N1 and N2 targets and viral genomic material concentrations as variables. The equation obtained was used for viral load calculations for each set of clinical samples, finally expressed as an average of the values for N1 and N2 targets. Regression coefficients over 0.99 were obtained for the viral load calibration curves. The RT-qPCR method used in this study has a high sensitivity of 97.7% and a low LoD of 5 copies/uL (Freire-Paspuel et al., 2020; Freire-Paspuel and Garcia-Bereguiain, 2021).

### Statistical Analysis

We carried out a descriptive study of the characteristics of the population by sex, age, and presence or absence of symptoms. The sensitivity, specificity, positive predictive value, and negative predictive value of the three different commercial brands of lateral flow immunochromatography based SARS-CoV-2 Rapid Diagnosis Antigen tests (RDAgT) were calculated in the general population, separating them into symptomatic and asymptomatic individuals at two different laboratories with a confidence level of 95%. Furthermore, Sensitivity and Negative Predictive Values (NPV) for different viral load detection thresholds of Limit of Detection (LoD) by RT-qPCR are presented.

All statistical analysis was carried out using SPSS Statistics 23 software.

## Results

A descriptive analysis was performed by age, sex, and presence or absence of symptoms in the total study population (**Table 1**). Most of the population was female (618/1076, 57.4%) and the highest number of participants ranged in age from 20 – 40 years (593/1076, 55.1%). It should be noted that sex and age information from one of the laboratories is not included as it was not collected. Conversely, the distribution of individuals according to the presence or absence of symptoms is provided for the whole population study, with a greater number of asymptomatic patients (1119/1646, 68%), as detailed in **Table 1**.

**Table 1:** Characteristics of the population tested with the three different commercial brands of lateral flow immunochromatography based SARS-CoV-2 Rapid Diagnosis Antigen tests (RDAgT) included in this study.

Brand	Total samples	Age (years)			Sex		Symptoms	
		≤ 20	20 and 40	≥40	Female	Male	Symptomatic	Asymptomatic
**Rapigen**	200	5 (6,8%)	122 (20,6%)	73 (17,8%)	116 (18,8%)	84 (18,3%)	138 (26,2%)	62 (5,5%)
**Certest**	653	46 (63%)	358 (60,4%)	249 (60,7%)	372 (60,2%)	281(61,4%)	166 (31,5%)*	1057 (94,5%)*
**SD-Biosensor**	223	22 (30,1%)	113 (19,1%)	88 (21,5%)	130 (21%)	93 (20,3%)	223 (42,3%)	0
**Total**	1076	73 (6,8%)	593 (55,1%)	410 (38,1%)	618 (57,4%)	458 (42,6%)	527 (32%)	1119 (68%)

*For CerTest, the information on gender and age does not include data from OneLabt laboratory in Ballenita, Santa Elena province, Ecuador.

### Overall Clinical Performance for the Three SARS-CoV-2 Rapid Diagnosis Antigen Tests Included in the Study

The analysis of the clinical performance for Rapigen, SD-Biosensor, and Certest RDAgTs is detailed in **Table 2**. The number of samples tested was 200, 223, and 1,223 for Rapigen, SD-Biosensor, and Certest, respectively. The ratio values for the number of positive SARS-CoV-2 samples by each RDAgT compared to RT-qPCR were 44/96, 98/124, and 102/159 for Rapigen, SD-Biosensor, and Certest, respectively. So, the overall sensitivity values for the RDAgTs evaluated in the present work were 45.8% (IC95%: 35.8 - 55.8), 79% (IC95%: 72 - 86.2), and 64.2% (IC95%: 56.7 - 71.6) for Rapigen, SD-Biosensor, and Certest, respectively (**Table 2**).

**Table 2:** Clinical performance of the three different commercial brands of lateral flow immunochromatography based SARS-CoV-2 Rapid Diagnosis Antigen tests (RDAgT) included in this study (total samples: number of samples included in the evaluation; positive samples: number of SARS-CoV-2 positive samples included in the evaluation for RDAgTs or RT-qPCR; negative samples: number of SARS-CoV-2 negative samples included in the evaluation for RDAgTs or RT-qPCR; PPV, positive predictive value; NPV, negative predictive value; parentesis includes IC95%.

RDAgT brand	Total samples	Positive samples (RDAgT/RT-qPCR)	Negative samples (RDAgT/RT-qPCR)	Sensitivity (%)	Specificity (%)	PPV (%)	NPV (%)
**Rapigen**	200	44/96	104/104	45.8 (35.8 - 55.8)	100	100	66.7 (59.2 - 74)
**CerTest**	1,223	102/159	1,040/1,064	64.2 (56.7 - 71.6)	97.7 (96.8 - 98.6)	81 (74.1 – 87.85)	94.8 (93.5 - 96.1)
**SD-Biosensor**	223	98/124	99/99	79 (72 – 86.2)	100	100	79.2 (72 - 86.3)

No SARS-CoV-2 false-positive samples were found for RDAgTs from the Rapigen and SD-Biosensor brands, so the specificity in both cases was 100%. For the Cerstest RDAgT, a total of 1,040 SARS-CoV-2 negative samples out of 1,064 samples were correctly identified, yielding a specificity of 97.7% (**Table 2**).

### Evaluation of the Analytical Sensitivity for the Three SARS-CoV-2 Rapid Diagnosis Antigen Tests Included in the Study

In **Table 3**, the analysis of the clinical performance at different limit of detection (LoD) or viral load thresholds for the three RDAgTs evaluated in this study is detailed. The values of LoD for which the sensitivity is over 90% were as follows: 100 copies/uL for Certest (90.8%, IC95%: 85.4 - 96.2), 1,000 copies/uL for SD-Biosensor (94.7%, IC95% 90.2 – 99.2), and 1,000,000 copies/uL for Rapigen (100%). For an LoD of 1,000,000 copies/uL, the sensitivity values for Certest and SD-Biosensor were 100% and 97.4% (IC95%:92.4 – 100), respectively.

**Table 3:** Evaluation of the analytical sensitivity of the three different commercial brands of lateral flow immunochromatography-based SARS-CoV-2 Rapid Diagnosis Antigen tests (RDAgT) included in this study.

LoD (copies/mL)	Rapigen			CerTest Biotec			SD-Biosensor		
	N	Sensitivity (%)	% NPV	N	Sensitivity (%)	% NPV	N	Sensitivity (%)	%NPV
**10^2^**	44/76	57.9 (46.8 - 69)	76.4 (69.3 - 83.6)	99/109	90.8 (85.4 - 96.2)	99 (98.5 - 99.6)	96/108	88.8 (82.8 – 94.7)	84.6 (77.9 – 91.3)
**10^3^**	42/66	63.6 (51.2 - 75.2)	81.2 (74.5 - 88)	86/91	94.5 (89.8 - 99.1)	99.5 (99.1 - 99.9)	90/95	94.7 (90.2 – 99.2)	95 (90.8 - 99.2)
**10^4^**	38/50	76 (64.1- 87.8)	89.6 (84.1 - 95.2)	57/59	96.6 (92 - 100)	99.8 (99.5 - 100)	83/86	96.5 (92.6 – 100)	97 (93.7 – 100)
**10^5^**	29/33	87.9 (76.8 - 99)	96.3 (92.7 – 99.8)	33/34	97.1 (91.3 - 100)	99.9 (99.7 - 100)	64/65	98.5 (95.5 – 100)	99 (97 -100)
**10^6^**	11/11	100	100	10/10	100	100	38/39	97.4 (92.4 – 100)	99 (97 – 100)

Sensitivity and Negative Predictive Values (NPV) for different viral load detection thresholds of Limit of Detection (LoD) by RT-qPCR are presented next to the 95% confidence interval.

**Figure 1:**
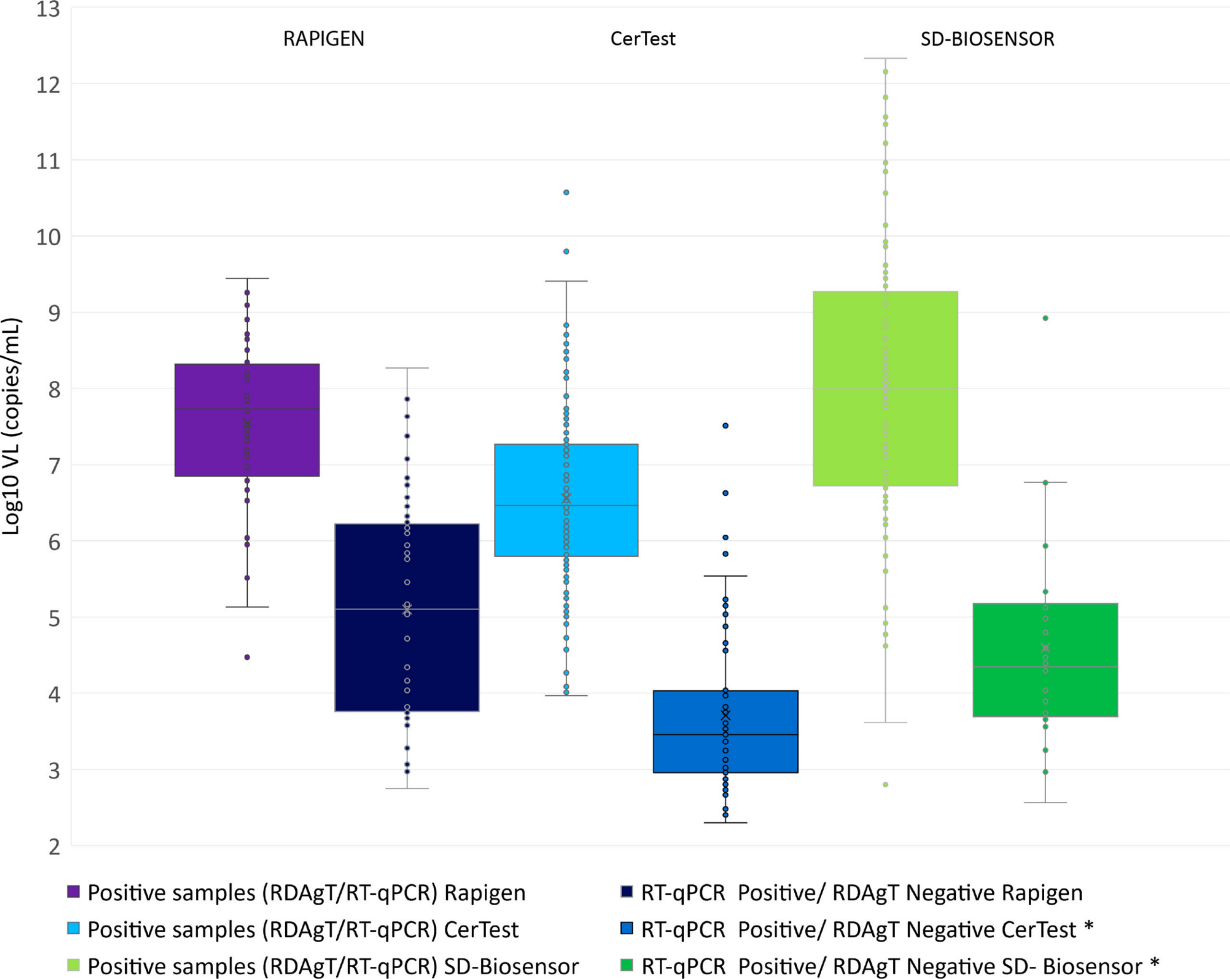
Viral loads distribution for all the SARS-CoV-2 positive samples by RT-qPCR was included in the study. Viral loads (VL) are presented on a Log10 scale. The different sets of samples used for each Rapid Diagnosis Antigen Test (RDAgT) brand are divided into two categories: RDAgT positive and RDAgT negative. *There are statistically significant differences (p < 0.05) for VL between RDAgT positive and RDAgT negative only for Certest Biotec and SD-Biosensor brands.

In **Figure 1**, the viral load distribution for the SARS-CoV-2 positive samples by RT-qPCR included in each RDAgTs evaluation is detailed. There are statistically significant differences (p < 0.05) for the mean viral load between RDAgT positive and RDAgT negative samples for Certest and SD-Biosensor, but not for Rapigen.

### Clinical Performance for the Three SARS-CoV-2 Rapid Diagnosis Antigen Tests Included in the Study for Symptomatic and Asymptomatic Individuals

In **Table 4**, the clinical performance of the three RDAgTs in symptomatic and asymptomatic individuals is shown. For Rapigen, the sensitivity values for symptomatic and asymptomatic individuals were 48.7% and 28.6%, respectively. For Certest, the sensitivity values for symptomatic and asymptomatic individuals were 79.7% and 52.2%, respectively. For SD-Biosensor, the sensitivity values were only addressed for symptomatic individuals, as asymptomatic individuals were excluded in this evaluation, so the value and overall sensitivity (79%) are the same as reported above. In **Table 4** we included a sensitivity value of 43.6% that has been reported in another study (Weitzel et al., 2021) for SD-Biosensor with asymptomatic individuals for comparison. There was a significant reduction in sensitivity (p < 0.05) for asymptomatic individuals compared to symptomatic individuals for the Certest and Rapigen RDAgTs.

**Table 4:** Clinical performance of the three different commercial brands of lateral flow immunochromatography based SARS-CoV-2 Rapid Diagnosis Antigen tests (RDAgT) included in this study for symptomatic and asymptomatic individuals (total samples: number of samples included in the evaluation; positive samples: number of SARS-CoV-2 positive samples included in the evaluation for RDAgTs or RT-qPCR; negative samples: number of SARS-CoV-2 negative samples included in the evaluation for RDAgTs or RT-qPCR; PPV: positive predictive value; NPV negative predictive value; next to the 95% confidence interval (IC95%).

Type of individual	RDAgT Brand	Total samples	Positive samples (RDAgT/RT-qPCR)	Negative samples (RDAgT/RT-qPCR)	Sensitivity (%)	Specificity (%)	PPV (%)	NPV (%)
**Symptomatic**	Rapigen	138	40/82	56/56	48.7 (37.8 – 59.5)	100	100	57.1 (47.3 – 66.9)
	Certest	166	55/69	94/97	79.7 (70.2 – 89.2)	97 (93.6 – 100)	94.8 (89.1-100)	87 (80.6- 93.3)
	SD-Biosensor	223	98/124	99/99	79 (71.8 – 86.2)	100	100	79.2 (72.1- 86.3)
**Asymptomatic**	Rapigen	62	4/14	48/48	28.6 (5 – 52.3)	100	100	82.8 (73.1-92.5)
	CerTest	1057	47/90	946/967	52.2 (41.9 – 62.5)	97.8 (96.8 – 98.7)	69.1 (58.1-80.1)	95.6 (94.3-96.9)
	SD-Biosensor*	286	44/101	178/185	43.6 (33.5 – 52.9)	96.2 (93.4 – 98.9)	86 (76.5 – 95.5)	75.7 (70.2- 81.2)

*For SD-Biosensor, as non-asymptomatic individuals were included in our study, we took values from the published report described in reference 26).

### Comparison of the Clinical Performance for the Rapid Diagnosis Antigen Test From Certest at Two Different Laboratories Located in Quito (Pichincha Province, Andean Region of Ecuador) and Ballenita (Santa Elena Province, Coastal Region of Ecuador)

For the clinical performance evaluation of Certest RDAgTs, there were two independent laboratories involved in the evaluation. In **Table 5**, the results of the clinical performance of the RDAgTs are presented for each of those two labs. In the UDLA lab, 653 samples were processed and the values for sensitivity and specificity were 72.2% and 95.9%, respectively. In the Onelabt laboratory, 653 samples were processed and the values for sensitivity and specificity were 57.5% and 100%, respectively.

**Table 5:** Independent evaluation of the clinical performance of the SARS-CoV-2 Rapid Diagnosis Antigen Test (RDAgT) from Certest Biotec at two different laboratories in Ecuador (total samples: number of samples included in the evaluation; positive samples: number of SAS-CoV-2 positive samples included in the evaluation for RDAgTs or RT-qPCR; negative samples: number of SARS-CoV-2 negative samples included in the evaluation for RDAgTs or RT-qPCR; PPV, positive predictive value; NPV, negative predictive value; parentesis includes IC95%.

Clinical Lab	Total samples	Positive samples (RDAgT/RT-qPCR)	Negative samples (RDAgT/RT-qPCR)	Sensitivity (%)	Specificity (%)	PPV (%)	NPV (%)
**UDLA**	653	52/72	581/605	72.2 (61.9 – 82.5)	95.9 (94.3 – 97.5)	68.4 (57.9 – 78.8)	96.5 (95 – 97.57)
**OneLabt**	570	50/87	483/483	57.5 (47.1-67.9)	100	100	92.9 (90.7 – 95.1)

## Discussion

In this study, we describe the clinical performance of three commercial RDAgTs brands currently available in several South American countries, including Ecuador. We found differences in terms of the overall sensitivity for the three RDAgTs evaluated. While Rapigen has a substantially reduced sensitivity below 50%, Certest and SD-Biosensor have an equivalent performance of almost 80% sensitivity for symptomatic individuals. Moreover, for a viral load threshold of 100 copies/uL, only the Certest RDAgT had an overall sensitivity over 90%. Both Certest and SD-Biosensor had sensitivity values close to 95% when samples with viral loads lower than 1000 copies/uL were excluded from the analysis. However, the overall sensitivity of Rapigen only reached a value over 90% for samples with viral loads over 10^6^ copies/uL. As an approximate LoD of 10^6^ copies/ml has been proposed as the minimal analytical sensitivity by the WHO or the Department of Health and Social Care from the United Kingdom (Department of Health and Social Care, 2020; WHO & R&D Blue Print, 2020), only SD-Biosensor and Certest RDAgTs evaluated in this study accomplished that requirement. Moreover, as the viral load is a dynamic parameter that may grow exponentially during the incubation period, our results would support the use of either Certest or SD-Biosensor over Rapigen RDAgTs (Avanzato et al., 2020; Kawasuji et al., 2020; Kleiboeker et al., 2020; Lavezzo et al., 2020; Pekosz et al., 2021; Singanayagam et al., 2020; Walsh et al., 2020; Weiss and Bellmann-Weiler, 2021).

Additionally, we call attention to the variability of sensitivity and specificity among the two labs involved in this evaluation study. As the same protocol for sample collection and RT-qPCR was used in both laboratories, the differences observed in sensitivity were associated at a random event such as a higher number of individuals with low viral loads in one of the locations. This difference in sensitivity occurred considering that more than 500 samples were evaluated in each lab setting, pointing out the need for extensive and multi-center studies for an accurate clinical performance evaluation of commercial RDAgTs. As reflected in **Table 6**, our results are within the range of sensitivity and specificity reported for RDAgTs, but there are substantial differences in the clinical performance between the different studies, even for the same RDAgT commercial brand (Albert et al., 2021; Baro et al., 2021; Cerutti et al., 2020; Corman et al., 2021; Cubas-Atienzar et al., 2021; Iglὁi et al., 2021; Lee et al., 2021; Nagura-Ikeda et al., 2020; Pérez-García et al., 2021; Pray et al., 2021; Pollock et al., 2021; Tinker et al., 2021). Moreover, the vast majority of clinical performance evaluations for RDAgTs have been carried out in high-income countries. However, SARS-CoV-2 current transmission is also happening in middle- and low-income countries where COVID-19 vaccination programs are progressing slowly. Moreover, SARS-COV-2 genomic surveillance in developing countries is limited, so new mutations or SARS-CoV-2 variants may not be well characterized. Under this scenario, locally assessed studies of the available RDAgT commercial brands are needed, as there is a concern regarding the potential reduction of sensitivity for SARS-CoV-2 variants (Frediani et al., 2021).

**Table 6:** Comparative analysis of the clinical performance for several SARS-CoV-2 Rapid Diagnosis Antigen Test (RDAgTs) with evaluation studies published in peer review journals (* for Abbott, results from two different commercial RDAgTs are included).

RDAgT Brand	Sensitivity (%)	Specificity (%)	Reference
**CerTest (Spain)**	53.5-79.7	97.7-100	14,21,27, our study.
**Rapigen (South Korea)**	28.6-62	100	14,20,25, our study.
**SD-Biosensor (South Korea)**	43.6-79	96.2-100	14,21,26,27, our study
Abbott (USA)*	20-79.6	100	14,17,18,21,27

In terms of specificity, the three RDAgTs showed a good performance with values of 100% for Rapigen, SD-Biosensor, and also for Certest at one of the laboratories. Interestingly, there was almost a 5% reduction in specificity for the Certest RDAgT only for the UDLA lab evaluation. It is important to note that the two labs involved in the study were located in a tropical latitude (Ecuador) but were two environmentally different settings: Quito is in the Andean Region of Ecuador at 2800 meters above sea level and Ballenita is at sea level in the Santa Elena province in the coastal region of Ecuador. As the weather conditions are different among these two locations, cross reactivity with a respiratory virus circulating at the time of this study is a plausible explanation for the differences observed between Quito and Ballenita. A similar phenomenon has been described for anti-SARS-CoV-2 serological tests, particularly in developing countries and tropical regions, due to the higher prevalence of some pathogens compared to high-income countries, where most of the COVID-19 diagnosis tools evaluations are conducted (Cota et al., 2020; Tso et al., 2021; Echeverría et al., 2021). Our results endorse the need for locally assessed evaluation studies in middle- and low-income settings to guarantee a reliable specificity for SARS-CoV-2 detection with RDAgTs.

This clinical performance evaluation has some limitations. For instance, no viral cultures were used to assess the LoDs as no BSL3 facility was available. However, the viral load calculations made by using a tittering of the CDC-designed SARS-CoV-2 positive control, described in the methods, were in agreement with other reports analyzing the same commercial brand RDAgTs (Corman et al., 2021; Lee et al., 2021; Mina et al., 2020; Pérez-García et al., 2021; Weitzel et al., 2021). Another limitation is that SD-Biosensor only included symptomatic patients, although this commercial brand is among the most used worldwide and several evaluation reports have already been published (Cubas-Atienzar et al., 2021; Lee et al., 2021). Moreover, the two laboratories were only involved in Certest RDAgT evaluation as the Ecuadorian representatives for the other two brands could not provide as many tests as requested.

In conclusion, the clinical performance and analytical sensitivity of Certest and SD-Biosensor RDAgT brands tested were within the WHO requirements. These results support the use of RDAgTs as a fast, cheap, and reliable point of care tool for SARS-CoV-2 detection for most COVID-19 contagious individuals. The massive use of RDAgTs would have a tremendous impact on COVID-19 pandemic control in developing countries where SARS-CoV-2 remains at a high level of transmission.

## Data Availability Statement

The original contributions presented in the study are included in the article/supplementary material. Further inquiries can be directed to the corresponding author.

## Ethics Statement

The studies involving human participants were reviewed and approved by IRB certified by Ministry of Health from Ecuador (code 008-2020). The patients/participants provided their written informed consent to participate in this study.

## Author Contributions

DM-J and MG-B wrote the manuscript. All the authors contributed to data collection and analysis, and also to manuscript revision and approval prior to submission.

## Funding

This study was funded by Universidad de Las Americas.

## Conflict of Interest

'Author FP is employed by OneLabt. The remaining authors declare that the research was conducted in the absence of any commercial or financial relationships that could be construed as a potential conflict of interest.

## Publisher’s Note

All claims expressed in this article are solely those of the authors and do not necessarily represent those of their affiliated organizations, or those of the publisher, the editors and the reviewers. Any product that may be evaluated in this article, or claim that may be made by its manufacturer, is not guaranteed or endorsed by the publisher.
